# An ATF_24_ peptide-functionalized β-elemene-nanostructured lipid carrier combined with cisplatin for bladder cancer treatment

**DOI:** 10.20892/j.issn.2095-3941.2020.0454

**Published:** 2020-08-15

**Authors:** Bingtao Zhai, Peng Chen, Wengang Wang, Shuiping Liu, Jiao Feng, Ting Duan, Yu Xiang, Ruonan Zhang, Mingming Zhang, Xuemeng Han, Xiaying Chen, Qiujie Li, Guohua Li, Ying Liu, Xingxing Huang, Wenzheng Zhang, Ting Pan, Lili Yan, Ting Jin, Tian Xie, Xinbing Sui

**Affiliations:** ^1^Department of Medical Oncology, the Affiliated Hospital of Hangzhou Normal University, College of Medicine, Hangzhou Normal University, Hangzhou 310018, China; ^2^Key Laboratory of Elemene Class Anti-cancer Chinese Medicine of Zhejiang Province and Engineering Laboratory of Development and Application of Traditional Chinese Medicine from Zhejiang Province, Hangzhou Normal University, Hangzhou 310018, China; ^3^College of Pharmacy, Chengdu University of Traditional Chinese Medicine, Chengdu 611137, China; ^4^Department of Medical Oncology, Sir Run Run Shaw Hospital, Zhejiang University, Hangzhou 310016, China

**Keywords:** uPAR, β-elemene, active targeting liposome, bladder cancer, DDP

## Abstract

**Objective:** In this study, we aimed to develop an amino-terminal fragment (ATF) peptide-targeted liposome carrying β-elemene (ATF_24_-PEG-Lipo-β-E) for targeted delivery into urokinase plasminogen activator receptor-overexpressing bladder cancer cells combined with cisplatin (DDP) for bladder cancer treatment.

**Methods:** The liposomes were prepared by ethanol injection and high-pressure microjet homogenization. The liposomes were characterized, and the drug content, entrapment efficiency, and *in vitro* release were studied. The targeting efficiency was investigated using confocal microscopy, ultra-fast liquid chromatography, and an orthotopic bladder cancer model. The effects of ATF_24_-PEG-Lipo-β-E combined with DDP on cell viability and proliferation were evaluated by a Cell Counting Kit-8 (CCK-8) assay, a colony formation assay, and cell apoptosis and cell cycle analyses. The anticancer effects were evaluated in a KU-19-19 bladder cancer xenograft model.

**Results:** ATF_24_-PEG-Lipo-β-E had small and uniform sizes (˜79 nm), high drug loading capacity (˜5.24 mg/mL), high entrapment efficiency (98.37 ± 0.95%), and exhibited sustained drug release behavior. ATF_24_-PEG-Lipo-β-E had better targeting efficiency and higher cytotoxicity than polyethylene glycol (PEG)ylated β-elemene liposomes (PEG-Lipo-β-E). DDP, combined with ATF_24_-PEG-Lipo-β-E, exerted a synergistic effect on cellular apoptosis and cell arrest at the G2/M phase, and these effects were dependent on the caspase-dependent pathway and Cdc25C/Cdc2/cyclin B1 pathways. Furthermore, the *in vivo* antitumor activity showed that the targeted liposomes effectively inhibited the growth of tumors, using the combined strategy.

**Conclusions:** The present study provided an effective strategy for the targeted delivery of β-elemene (β-E) to bladder cancer, and a combined strategy for bladder cancer treatment.

## Introduction

Cancer is a major cause of morbidity and mortality worldwide, regardless of the level of economic development. Worldwide, bladder cancer is the sixth most common cancer among males^1^. In China, bladder cancer is one the most common malignant tumors of the urinary system among males^2,3^. For patients with non-muscle-invasive bladder cancer (NMIBC), radical cystectomy with lymphadenectomy are the standard treatments^4^. In contrast to NMIBC, muscle-invasive bladder cancer (MIBC) is a poly-phase cancer that may metastasize to the prostate, vagina, or bowel, and has a much lower 5-year survival rate^5^. Thus, a better understanding of the molecular mechanisms responsible for the invasive behavior of bladder cancer is important.

The system consists of urokinase-type plasminogen activator (uPA)/urokinase-type plasminogen activator receptor (uPAR), which plays a critical role in fibrinolysis of the extracellular matrix, and facilitates tumor cell migration and invasion^6^. High uPAR expression has been detected in tumor tissues in patients with high metastatic cancer and a poor prognosis. In contrast, strategies that block uPAR expression decrease the invasive capacity of tumor cells^7–9^. Hau et al.^10^ further identified increased uPAR expression in 94% of invasive human bladder cancers and in 54%–71% of noninvasive bladder cancers. Agents that inhibit uPAR expression may therefore be useful for developing antitumor therapies.

In recent years, drug delivery systems using drug-loaded active targeting liposomes have attracted attention, with the aim of increasing the number of liposomes internalized into tumor cells^11^. Liposome targeting of only tumor cell surface targets can only be delivered into the tumor interstitial space *via* “leaky” tumor vasculatures mediated by the enhanced permeability and retention (EPR) effect^12,13^. Molecular targets allowing for active targeting to the tumor endothelial cell layer, tumor stroma, and tumor cells can facilitate the crossing of the endothelial layer by drug-loaded liposomes, thus increasing the efficiency of liposomes in tumor cells^14^. The uPAR is primarily expressed by myofibroblasts and macrophages in the tumor-associated stroma and some tumor cells^15,16^. The high level of uPAR expression in MIBC tissues supports the development of targeted liposome drug carriers for the effective treatment of MIBC. Studies have shown that an amino-terminal fragment (ATF) peptide can compete with uPA for the binding of uPAR at the surface of both tumor and endothelial cells, resulting in the inhibition of tumor growth and angiogenesis. For example, the delivery of uPAR-targeted iron oxide nanoparticles carrying gemcitabine or cisplatin (DDP) resulted in significant growth inhibition in pancreatic tumors^17–19^.

**Figure 1:**
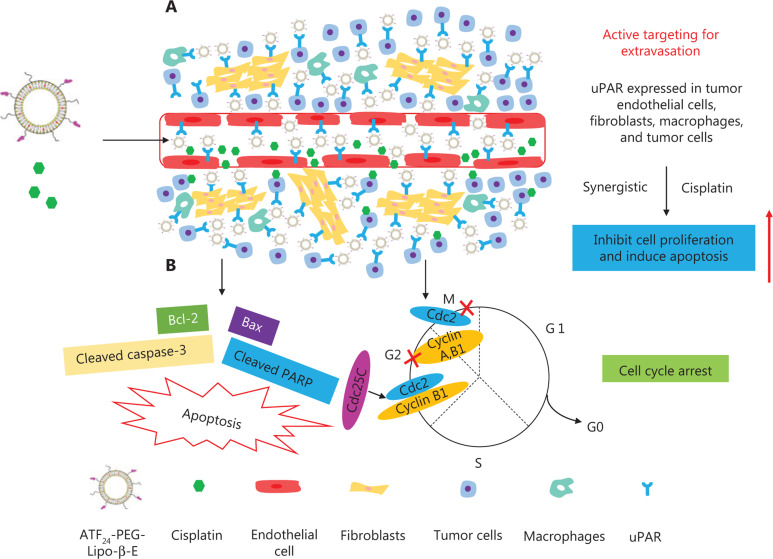
The proposed mechanism of urokinase-type plasminogen activator receptor (uPAR)-targeted β-E liposomes combined with cisplatin (DDP) for the treatment of bladder cancer. (A) The uPAR was highly expressed in tumor cells, angiogenic endothelial cells, stromal fibroblasts, and macrophages. The binding of ATF_24_ peptide-modified liposomes to stromal fibroblasts, macrophages, and tumor cells enhanced the retention of liposomes in the tumor microenvironment and tumor tissues. Receptor-mediated internalization of nanoparticle drug carriers increased cellular drug delivery and therapeutic effects. (B) The combination of ATF_24_-PEG-Lipo-β-E and DDP significantly inhibited KU-19-19 cell proliferation and promoted cell apoptosis, which were associated with the caspase-dependent pathway and the Cdc25C/Cdc2/cyclin B1 signaling pathway.

β-Elemene (β-E) is a broad-spectrum antitumor drug extracted from the traditional Chinese medicine, *Curcuma wenyujin* Y. H. Chen et C. Ling. To date, an elemene emulsion injection and an oral emulsion have been developed and used to treat various cancers for more than 20 years^20^. Studies have shown that these emulsions can be used to enhance the efficacy and reduce the toxicity of chemoradiotherapy, and reverse drug resistance without significant side effects^21–23^. To overcome the drawback of low delivery efficiency of conventional elemene emulsions, we developed a novel ATF_24_ peptide-targeted liposome carrying β-E (ATF_24_-PEG-Lipo-β-E) for targeted delivery into uPAR-expressing tumors and stromal cells, combined with DDP for bladder cancer treatment. The proposed mechanism of uPAR-targeted β-E liposomes combined with DDP for the treatment of bladder cancer is shown in **Figure 1**.

## Materials and methods

### Materials

The following reagents were obtained: ATF_24_ peptides (97.16% pure; ChinaPeptides, Shanghai, China), β-E crude drug (99.1% pure; Jusheng Technology, Hubei, China), DDP, Cell Counting Kit-8 (CCK-8), and DiD (1,1′-dioctadecyl-3,3,3′,3′-tetramethylindodicarbocyanine perchlorate) (Dalian Meilun Biotechnology, Dalian, China), DDP injection (Haosen Pharmaceutical Group, Jiangsu, China), acetonitrile (Merck, Darmstadt, Germany), soybean lecithin (Tywei, Shanghai, China), cholesterol (99.5% pure; Dingguo Changsheng Biotechnology, Beijing, China), Lipoid DSPE-PEG_2000_ (Lipoid, Ludwigshafen, Germany), DSPE-PEG_2000_-COOH (Avanti Polar Lipids, Alabaster, AL, USA), RPMI 1640 and fetal bovine serum (FBS) (Gibco, Waltham, MA, USA), DAPI (4′,6-diamidino-2-phenylindole) and Crystal Violet (Beyotime, Shanghai, China), ProLong™ Gold Antifade Reagent (Invitrogen, Carlsbad, CA, USA), a BD Pharmingen™ FITC Annexin V Apoptosis Detection Kit I (BD Biosciences, San Jose, CA, USA), and a Cell Cycle Staining Kit [Multi Sciences (Lianke) Biotech, Hangzhou, China].

### Cell culture

The human bladder cancer cells, RT-4 and KU-19-19 (obtained from the American Type Culture Collection, Manassas, VA, USA) were cultured in 5A medium and RPMI 1640 medium, respectively, at 37 °C in a 5% CO_2_ humidified environment incubator (Thermo Fisher Scientific, Waltham, MA, USA). The medium contained 10% FBS, 100 U/mL penicillin and 100 mg/mL streptomycin.

### Mice

Female nude mice (6–8 weeks old) were purchased from the Institute of Experimental Animals of the Zhejiang Academy of Medical Sciences (Hangzhou, China) and housed in a standard polypropylene cage containing sterile bedding under a controlled temperature (23 ± 2 °C) and humidity (50 ± 5%). All animal study protocols were approved by the Medical Ethics Committee of the Hangzhou Normal University (Approval No. 2017052).

### Conjugation of the ATF_24_ peptide with DSPE-PEG_2000_-COOH

The uPAR targeting peptide, ATF_24,_ was conjugated with DSPE-PEG_2000_-COOH by a dehydration condensation reaction to obtain the targeting compound, DSPE-PEG_2000_-ATF_24_. Briefly, the ATF_24_ peptide, DSPE-PEG_2000_-COOH, and hexafluorophosphate benzotriazole tetramethyl uronium were dissolved in newly distilled dimethylformamide (molar ratio: 1:3:3), and then a 10-fold higher amount of diisopropylethylamine was immediately added. After 50 min of incubation at room temperature under moderate stirring, the reaction mixture was dialyzed [molecular weight (MW) cutoff = 3,500 Da] against deionized water to remove the unconjugated peptide and reaction medium. The final solution was lyophilized and stored at −20 °C until use. Liquid chromatography-electrospray ionization-tandem mass spectrometric (LC-ESI-MS) (AB Sciex, Redwood City, CA, USA) was used to investigate the MW of the ATF_24_ peptide. The MW of DSPE-PEG_2000_-ATF_24_ was determined by a matrix assisted laser desorption/ionization-time of flight mass spectrometer (MALDI-TOF-MS) (Bruker Daltonics, Hamburg, Germany)^24^.

### Preparation of ATF_24_-PEG-Lipo-β-E

Ethanol injection and high-pressure microjet homogenization were used to prepare the polyethylene glycol (PEG)ylated liposomes and ATF_24_ modified liposomes. Specifically, soybean lecithin (2.5 g), cholesterol (0.1 g), and DSPE-PEG_2000_ (0.3 g) were dissolved in 2 mL of 95% ethanol in an 80 °C water bath. The oil phase was slowly added to 100 mL of water containing 10 mM L-histidine (pH 6.5) at 60 °C, followed by stirring using an Ultra-Turrax T18 high-speed blender (IKA, Staufen, Germany) at 13,700 × *g* for 60 min. The solution was passed through an LM20 microfluidizer (Microfluidics, Westwood, MA, USA) at a pressure of 15,000 psi for 3 cycles, and then the PEGylated liposomes were formed^25^. The ATF_24_-modified liposomes were prepared with 0.3 g of DSPE-PEG_2000_ in blank PEGylated liposomes (PEG-Lipo) being replaced by 0.2 g of DSPE-PEG_2000_-ATF_24_ and 0.1 g of DSPE-PEG_2000_ in a similar manner. The DiD-labeled and β-E-loaded liposomes were prepared by adding 5 mg of DiD and 0.5 g β-E, respectively, to the original formula.

### Characterization of ATF_24_-PEG-Lipo-β-E

The morphology of ATF_24_-PEG-Lipo-β-E was determined using transmission electron microscopy (TEM) (HT-7700; Hitachi, Tokyo, Japan). The mean particle size, zeta potential, and polydispersity index (PDI) of the liposome droplets were determined using a Zetasizer Nano ZS (Malvern Instruments, Malvern, UK)^26^. The pH of the samples was measured using a pH meter at 20 ± 1 °C (PB-10; Sartorius, Goettingen, Germany). The drug content was detected by ultra-fast liquid chromatography (UFLC) (Shimadzu, Kyoto, Japan). The entrapment efficiency (EE) of β-E in the ATF_24_-modified liposomes was determined by the liquid surface method^27^.

### Fourier transform infrared spectroscopy (FTIR) and differential scanning calorimetry (DSC)

The FTIR spectra of the pure drug, physical mixture (β-E/soybean lecithin/cholesterol/DSPE-PEG_2000_), PEG-Lipo, PEGylated β-elemene liposome (PEG-Lipo-β-E), and ATF_24_-PEG-Lipo-β-E were obtained using an FTIR spectrophotometer (ALPHA; Bruker, Bremen, Germany). The liposomes were freeze-dried before the measurement (ALPHA 1-2 LD plus; Christ, Frankfurt, Germany). The spectra were obtained in the wave number region between 4,000 and 400 cm^−1^ ^28,29^.

A thermal analysis of the ATF_24_-PEG-Lipo-β-E was performed using a differential scanning calorimeter (TA Q200; SelectScience, Bath, UK). The samples were analyzed at a heating rate of 5 °C/min from 0 to 80 °C^30^.

#### *In vitro* release of β-E from the ATF_24_-modified liposomes

Measurement of the* in vitro* release of free β-E, PEG-Lipo-β-E, and ATF_24_*-*PEG-Lipo-β-E was conducted by using the dialysis bag method. Briefly, 5 mL of β-E, PEG-Lipo-β-E or ATF_24_-PEG-Lipo-β-E (5 mg/mL) was placed in a dialysis membrane (MW cutoff: 300,000 Da), ligated, immersed in the 75% ethanol release media (100 mL), and stirred using a magnetic force at 37 °C. One mL of the sample was removed at predetermined time intervals of 0.5, 1, 2, 4, 6, and 8 h and diluted to 10 mL. The supernatants were filtered through 0.22 μm microporous membranes and subjected to UFLC analysis for the determination of β-E^25^.

#### The receptor expression levels

RT-4 and KU-19-19 cells seeded in 6-well plates were harvested and lysed in cell lysis buffer containing protease inhibitors. Equal amounts of total protein were loaded onto 12% sodium dodecyl sulfate-polyacrylamide gel electrophoresis (SDS-PAGE) gels for separation, transferred onto polyvinylidene difluoride (PVDF) membranes (Millipore, Billerica, MA, USA), and incubated with anti-uPAR (1:200; Santa Cruz Biotechnology, Santa Cruz, CA, USA) and anti-glyceraldehyde 3-phosphate dehydrogenase (GAPDH) (1:1,000; Cell Signaling Technology, Beverly, MA, USA) primary antibodies at 4 °C overnight. The membranes were washed with Tris-buffered saline containing 0.1% Tween 20 (TBST) solution. Then, the membranes were incubated with horseradish peroxidase-labeled secondary antibodies and detected with the ECL^™^ Prime Western Blotting Detection Reagent (GE Healthcare, Chicago, IL, USA) using a Bio-Rad ChemiDoc^™^ Imaging System (Bio-Rad Laboratories, Hercules, CA, USA).

#### Cellular uptake and in vitro cell migration

##### Cellular uptake

For qualitative analysis, 2 × 10^5^ KU-19-19 cells were seeded on round cell slides in 12-well plates and cultured for 24 h. The cells were incubated with the DiD-labeled liposomes (5 μg/mL) for another 4 h and washed twice with ice-cold phosphate-buffered saline (PBS). Then, the cells were fixed with 4% paraformaldehyde for 20 min and stained with 4′,6-diamidino-2-phenylindole for 5 min. The cells were then sealed using the ProLong™ Gold Antifade reagent. Finally, the cells were imaged using confocal laser scanning microscopy (LSM 710 NLO; Carl Zeiss Meditec, Dublin, CA, USA)^31^.

For quantitative analysis, KU-19-19 cells at a density of 1 × 10^6^ per well were seeded in 10 cm cell culture dishes and cultured for 24 h. The β-E, PEG-Lipo-β-E, and ATF_24_-PEG-Lipo-β-E were added to the culture media at a β-E concentration of 50 μg/mL, and incubated at 37 °C for 4 h. Then, the cells were trypsinized with a 0.25% trypsin-PBS solution and washed twice with ice-cold PBS, followed by a 3-cycle freeze-thaw procedure. Next, 600 μL of acetonitrile were added to 200 μL of the cell suspension with mixing for 10 min to precipitate the protein. After the centrifugation (9,400 × *g* for 10 min), the supernatant was collected, and the amount of β-E internalized by the KU-19-19 cells was detected using UFLC^32^.

##### *In vitro* cell migration

For the wound healing assay, the cells were seeded at a density of 2 × 10^6^ cells per well in 6-well plates and cultured until 80%–90% confluency was reached. Then, a sterile 200 μL pipette tip was used to generate a vertical wound. The cells were washed with PBS 3 times and incubated with PEG-Lipo, PEG-Lipo-β-E, or ATF_24_-PEG-Lipo-β-E (at an equivalent β-E dose of 45 μg/mL). Cells that did not receive any treatment after the scratch were used as a negative control. In the ATF_24_ + ATF_24_-PEG-Lipo-β-E group, the cells were incubated with excess free ATF_24_ for 30 min after scratching. Then, ATF_24_-PEG-Lipo-β-E was added to the cells, and the following processes were performed as described for the other groups. Images were captured at 0 and 36 h after the treatment using an inverted microscope (Carl Zeiss, Jena, Germany)^33^.

For the Transwell migration assay, 2 × 10^5^ cells were seeded in the upper chamber of Transwell plates (24-well insert, pore size: 8 μm; Corning, Corning, NY, USA). In total, 100 μL of serum-free media with PEG-Lipo, PEG-Lipo-β-E, or ATF_24_-PEG-Lipo-β-E (at an equivalent β-E dose of 45 μg/mL) was added to the upper chamber, while 600 μL of complete medium containing 10% FBS was loaded into the lower chamber as a chemoattractant. Cells without any treatment were used as controls. In the ATF_24_ + ATF_24_-PEG-Lipo-β-E group, the cells were incubated with excess free ATF_24_ for 30 min before adding the ATF_24_-PEG-Lipo-β-E. After 36 h, the upper medium was removed, and a cotton swab was used to remove the remaining cells. Then, the Transwell chambers were lightly washed with PBS, fixed using 4% paraformaldehyde for 20 min, and stained with Crystal Violet for 2 h. Finally, the upper chambers were washed with PBS twice, and the stained Transwell chambers were visualized using a microscope (Eclipse Ci-S; Nikon, Tokyo, Japan)^32^.

#### *In vivo* image studies

Female nude mice were injected with KU-19-19 cells to establish an orthotopic bladder cancer model. After 7 days of tumor establishment, the tumor-bearing mice were randomly assigned to 3 groups and received an intravenous injection of DiD, DiD-labeled PEGylated liposomes (DiD-PEG-Lipo), or DiD-labeled ATF_24_ peptide-targeted liposomes (DiD-ATF_24_-PEG-Lipo). After anesthetization with 1% pentobarbital sodium, the mice were imaged with an IVIS® Lumina LT Series III *in vivo* imaging system (PerkinElmer, Waltham, MA, USA) at 24, 48, and 60 h after injection. Then, the mice were sacrificed, and the hearts, livers, spleens, lungs, kidneys, and tumors were collected. All organs were also imaged with the IVIS® Lumina LT Series III *in vivo* imaging system (PerkinElmer)^31^.

#### *In vitro* cytotoxicity studies

To determine the cell viability after incubation with PEG-Lipo-β-E or ATF_24_-PEG-Lipo-β-E and DDP, a CCK-8 assay was carried out using the KU-19-19 cells. The cells were seeded at a density of 3×10^4^ cells per well in 96-well plates and incubated at 37 °C and 5% CO_2_ for 24 h. Then, various concentrations of drugs were added to the wells. The control was RPMI 1640 medium without drugs. After 48 h of incubation, 10 μL of CCK-8 reagent was added to each well. After 2 h of incubation, the absorbance measurements were performed at 450 nm using a microplate reader (Multiskan™ FC; Thermo Fisher Scientific, Waltham, MA, USA)^34^.

#### The effect of ATF_24_-PEG-Lipo-β-E combined with DDP on cell viability and proliferation

#### Cell viability assay

The cells were treated with ATF_24_-PEG-Lipo-β-E (40 or 45 μg/mL) and/or DDP (1 or 2 μg/mL) for 48 h. The other steps were the same as described above. The combination index (CI) was measured by using CompuSyn software (ComboSyn, Paramus, USA).

#### Colony formation assay

The cells were first digested with 0.25% trypsin and then divided into individual cells. Subsequently, 5,000 cells were seeded into 10 cm culture dishes, grown overnight, and treated with ATF_24_-PEG-Lipo-β-E (8 μg/mL) and/or DDP (0.2 μg/mL) for 12 days. Once the colonies were visible to the naked eye, the culture dish was washed twice with PBS. Then, the colonies were fixed with 4% paraformaldehyde for 20 min, followed by staining with Crystal Violet for 2 h. Images were captured using a digital camera (EOS 5D Mark IV; Canon, Tokyo, Japan)^35^.

#### Cell apoptosis analysis

Cell apoptosis was assessed by flow cytometry using a CytoFLEX S (Beckman Coulter Biotechnology, Suzhou, China). Briefly, following the treatment with ATF_24_-PEG-Lipo-β-E (45 μg/mL) and/or DDP (1 μg/mL) for 48 h, the cells (3 × 10^5^ cells/mL) were harvested and resuspended in 1× Annexin V-binding buffer. Subsequently, 4 μL of Annexin V-FITC and propidium iodide (PI) were added, and the cells were incubated for 10 min away from light at room temperature. Cell apoptosis was then analyzed using CytExpert software (Beckman Coulter, Brea, CA, USA)^35,36^.

#### Cell cycle analysis

Following treatment with ATF_24_-PEG-Lipo-β-E (45 μg/mL) and/or DDP (1 μg/mL) for 48 h, the cells (3 × 10^5^ cells/mL) were collected, washed twice with ice-cold PBS, suspended in 300 μL of Reagent A DNA Staining Solution and 3 μL of Reagent B Permeabilization Solution, and incubated for 30 min at 37 °C in the dark. Cell cycle analysis was performed using CytExpert software (Beckman Coulter)^37^.

#### Western blot analysis

The cells were treated with ATF_24_-PEG-Lipo-β-E and/or DDP for the indicated times. The PVDF membranes were incubated with anti-cyclin B1 (1:1,000, Cell Signaling Technology, anti-Bcl-2 (1:1,000; Cell Signaling Technology), anti-Bax (1:1,000; Cell Signaling Technology), anti-cleaved PARP (1:1,000; Cell Signaling Technology), anti-Cdc25C (1:200; Santa Cruz Biotechnology, Dallas, TX, USA), anti-Cdc2 p34 (1:200; Santa Cruz Biotechnology), anti-cleaved caspase-3 (1:400; Abcam, Cambridge, UK), and anti-GAPDH (1:1,000; Cell Signaling Technology) primary antibodies at 4 °C overnight. The other steps were the same as described above^38^.

### *In vivo* antitumor efficacy

The antitumor efficacy of ATF_24_-PEG-Lipo-β-E and the combined treatment of ATF_24_-PEG-Lipo-β-E and DDP injection were evaluated in tumor-bearing xenografts. In total, 25 female nude mice were used in this experiment, and each mouse was subcutaneously injected with a suspension of 5 × 10^5^ KU-19-19 human bladder cancer cells in PBS (100 μL). When the tumor volume (TV) reached ˜100 mm^3^, the mice were randomly divided into 5 groups (*N* = 5) and subjected to one of the following treatments: (a) PEG-Lipo, (b) PEG-Lipo-β-E (25 mg/kg), (c) ATF_24_-PEG-Lipo-β-E (25 mg/kg) *via* intravenous injection daily, (d) DDP injection (5 mg/kg), or (e) ATF_24_-PEG-Lipo-β-E (25 mg/kg) + DDP injection (5 mg/kg) intravenously twice^39^. The body weights were recorded, and tumor growth was monitored every 4 days. Sixteen days later, all animals were sacrificed by cervical dislocation, and the tumors were isolated and weighed. The TV was calculated by the following formula: TV = 0.5× (d1 × d2^2^), where d1 and d2 are the largest and the smallest perpendicular diameters, respectively. After the livers, spleens, and kidneys were harvested, hematoxylin and eosin (H&E) staining was used to evaluate the safety of the various formulations. Then, the tumor tissues were evaluated qualitatively to determine uPAR, Ki-67, and cleaved caspase-3 expressions using immunohistochemistry^40^.

### Statistical analysis

The data were statistically analyzed by Prism 7 software (GraphPad, San Diego, CA, USA). All analyses were conducted in triplicate, and the results are reported as the mean ± standard deviation (SD). A value of *P* < 0.05 was considered statistically significant (*P < 0.05; **P < 0.01; ***P < 0.001).

## Results

### Synthesis of DSPE-PEG_2000_-ATF_24_

**Figure 2:**
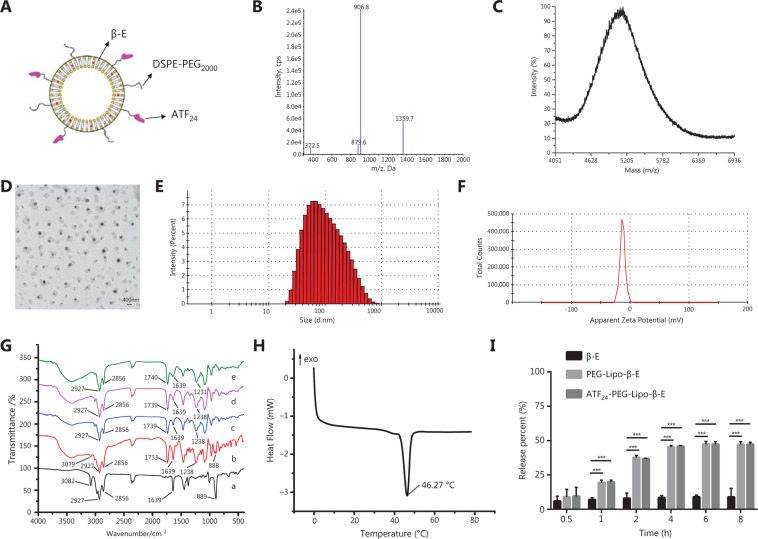
Characteristics of ATF_24_ modified PEGylated liposomes. (A) Schematic illustration of ATF_24_-PEG-Lipo-β-E. (B) LC-ESI-MS spectra of the ATF_24_ peptides. (C) MALDI-TOF-MS spectrum of DSPE-PEG_2000_-ATF_24_. (D) Morphology of ATF_24_-PEG-Lipo-β-E by TEM (2,000×). (E) Particle size of ATF_24_-PEG-Lipo-β-E. (F) Zeta potential of ATF_24_-PEG-Lipo-β-E. (G) FTIR spectrum of (a) β-E, (b) the physical mixture of (β-E/soybean lecithin/cholesterol/DSPE-PEG_2000_), (c) PEG-Lipo, (d) PEG-Lipo-β-E and (e) ATF_24_-PEG-Lipo-β-E. (H) Differential scanning chromatogram of ATF_24_-PEG-Lipo-β-E. (I) *In vitro* release behaviors of free β-E, PEG-Lipo-β-E and ATF_24_-PEG-Lipo-β-E in 75% ethanol at 37 °C. ***P < 0.001.

The ATF_24_ peptide Cys-Leu-Asn-Gly-Gly-Thr-Cys-Val-Ser-Asn-Lys-Tyr-Phe-Ser-Asn-Ile-His-Trp-Cys-Asn-Cys-Pro-Lys-Lys (purity > 97.16%) was synthesized according to standard solid phase peptide synthesis by ChinaPeptides (Shanghai, China)^19^. The theoretical MWs of ATF_24_ and DSPE-PEG_2000_-COOH were 2,717 and 2,750 Da, respectively^41^. As shown in **Figure 2B and 2C**, the MW of the ATF_24_ peptide as determined by LC-ESI-MS was 2,717 Da, and the MW of the reaction product as determined by MALDI-TOF-MS was approximately 5,143 Da, which was consistent with the theoretical MW of ATF_24_ and DSPE-PEG_2000_-ATF_24_, confirming that the synthetic product was the targeted compound.

### Preparation and characterization of ATF_24_-PEG-Lipo-β-E

A schematic diagram of ATF_24_-PEG-Lipo-β-E is shown in **Figure 2A**. DSPE-PEG_2000_-ATF_24_ (0.2%) was added to the liposomes to provide an active targeting effect. The TEM images generally displayed a spheroid shape with a size of 79.32 ± 1.282 nm (**Figure 2D and 2E**), and the PDI value was 0.28 ± 0.008, indicating a homogenous population of phospholipid vesicles. The zeta potential was −12.77 ± 0.416 mV, indicating a negatively charged surface on the liposomes (**Figure 2F**). The pH was 6.49 ± 0.017. The β-E content was 5.24 ± 0.362 mg/mL. The average EE of β-E in the ATF_24_-modified liposomes was 98.37 ± 0.95%.

### FTIR and DSC

The FTIR spectra obtained for the pure drug, physical mixture, and liposomes are shown in **Figure 2G**. In the spectra, the bands at 1739 and 1639 cm^−1^ indicated the presence of the C=C, C=O, and NH_2_ in the β-E, DSPE, soybean lecithin, and ATF_24_. The asymmetric stretching vibration of the P=O group was identified at 1238 cm^−1^ and 1231 cm^−1^ in the physical mixture and liposomes. In addition, the peaks at 2856 and 2927 cm^−1^ were related to the symmetric and asymmetric CH_2_ stretching vibrations in the aliphatic structure. The two peaks related to the β-E (3082^−1^ and 889 cm^−1^) were not observed in the spectra of PEG-Lipo-β-E and ATF_24_-PEG-Lipo-β-E likely because the drug was encapsulated in the liposomes. Moreover, the spectra of ATF_24_-PEG-Lipo-β-E were similar to those of PEG-Lipo and PEG-Lipo-β-E, indicating that the presence of the ATF_24_ peptide did not affect the formation of the liposomes^42^.

A thermogram of ATF_24_-PEG-Lipo-β-E is shown in **Figure 2H**. Our previous work showed that soybean lecithin, cholesterol, and a physical mixture of β-E/soybean lecithin/cholesterol/DSPE-PEG_2000_ each had a single endothermic peak at temperatures of 36.83 °C, 42.34 °C, and 45.11 °C, respectively. The DSC curve of ATF_24_-PEG-Lipo-β-E exhibited a high intensity sharp endothermic peak at 46.27 °C, which significantly differed from the peaks of the soybean lecithin, cholesterol, and the physical mixture. This peak was also detected in the thermograms of PEG-Lipo and PEG-Lipo-β-E at the temperatures of 45.99 °C and 46.14 °C, although the peak became less intense. These changes were attributed to a possible interaction between the liposomal components^42^.

### *In vitro* release of β-E from ATF_24_-modified liposomes

As shown in **Figure 2I**, PEG-Lipo-β-E and ATF_24_*-*PEG-Lipo-β-E exhibited a very slow release of β-E without an initial burst in 75% ethanol. Approximately 37% of the drug was released within the first 2 h; then, a sustained drug release profile was achieved in which 47% of the drug was released after 8 h, while free β-E displayed approximately 8.94% release within 8 h under these conditions. The release of β-E significantly increased after being encapsulated into the liposomes, but did not increase after the addition of ATF_24_, indicating that modification of the ATF_24_ peptide did not affect the release characteristics of the PEGylated liposomes.

### Receptor expression

**Figure 3:**
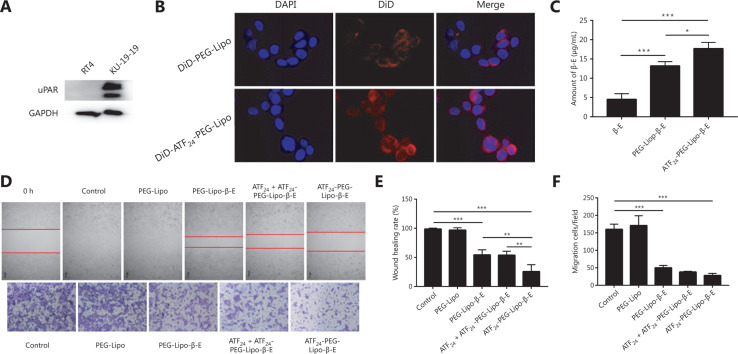
Cellular uptake and *in vitro* cell migration study. (A) The expression of urokinase-type plasminogen activator receptor (uPAR) in RT4 and KU-19-19 cells. (B) Confocal images of the 4 h cellular uptake of the DiD-labeled liposomes (DiD-PEG-Lipo and DiD-ATF_24_-PEG-Lipo) in KU-19-19 cells (60 ×). (C) Cellular uptake of free β-E, PEG-Lipo-β-E and ATF_24_-PEG-Lipo-β-E in KU-19-19 cells by ultra-fast liquid chromatography. (D) *In vitro* inhibitory effects of ATF_24_-PEG-Lipo-β-E on the cell migration abilities of KU-19-19 cells by a wound healing assay (20×) and Transwell migration assay (10×); cells were stained with Crystal Violet. (E) The quantified wound healing inhibitory effect of PEG-Lipo-β-E, ATF_24_ + ATF_24_-PEG-Lipo-β-E, and ATF_24_-PEG-Lipo-β-E. (F) Quantified cell migration inhibitory effects of PEG-Lipo-β-E, ATF_24_ + ATF_24_-PEG-Lipo-β-E, and ATF_24_-PEG-Lipo-β-E. *P < 0.05; **P < 0.01; ***P < 0.001.

The expression level of uPAR was detected using western blot analysis. As shown in **Figure 3A**, the highly invasive bladder cancer cell line, KU-19-19, had a higher expression level of uPAR than the less aggressive cell line, RT4.

### Cellular uptake study

To assess the cellular uptake and cancer-targeting ability of the ATF_24_-modified liposomes *in vitro*, KU-19-19 cells were cultured with DiD-PEG-Lipo or DiD-ATF_24_-PEG-Lipo. The ATF_24_-modified formulation showed a higher uptake than the PEGylated formulation, as shown in the fluorescent images of the KU-19-19 cell lines after incubation for 4 h (**Figure 3B**). The cellular uptakes of the free β-E, PEG-Lipo-β-E, and ATF_24_-PEG-Lipo-β-E were quantitatively evaluated by using UFLC. ATF_24_-PEG-Lipo-β-E displayed the highest cellular uptake. The uptake of free β-E was significantly lower than that of PEG-Lipo-β-E. Compared to the PEG-Lipo-β-E group, ATF_24_-PEG-Lipo-β-E increased the uptake amount of β-E by almost 1.34-fold (**Figure 3C**).

### *In vitro* cell migration

Because cell migration is closely related to tumor metastasis, the cell motility was examined using a wound healing assay. In the control group, the scratch gap was barely observed after 36 h, indicating that the KU-19-19 cells had strong motility. Blank liposomes without β-E did not affect cell motility. The PEG-Lipo-β-E groups prevented cell motility with 54.33% wound closure. The wound closure in the ATF_24_-PEG-Lipo-β-E group was reduced to only 25.67%, showing the highest inhibition of cell motility. After preincubation with ATF_24_, wound closure could not be efficiently suppressed by ATF_24_-PEG-Lipo-β-E (**Figure 3D and 3E**).

In the Transwell migration assay, PEG-Lipo had almost no inhibitory effect, but the inhibitory effect of PEG-Lipo-β-E was significant. Compared with the PEG-Lipo-β-E group, the migrated cells were further reduced in the ATF_24_-PEG-Lipo-β-E group. Moreover, after incubation with excess free ATF_24_ peptide, the migration of KU-19-19 cells could not be efficiently suppressed by ATF_24_-PEG-Lipo-β-E, which explained the specific binding of ATF_24_ and uPAR (**Figure 3D and 3F**). As shown in this figure, the liposomes decorated by ATF_24_ effectively inhibited tumor cell migration.

### *In vivo* image studies

**Figure 4:**
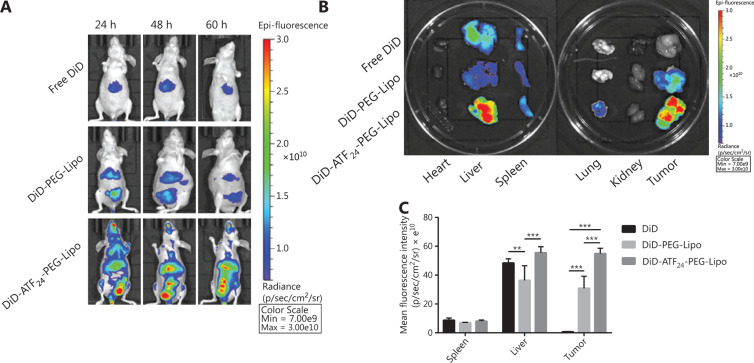
*In vivo* distribution of ATF_24_-PEG-Lipo and PEG-Lipo. (A) *In vivo* fluorescent images of KU-19-19 tumor-bearing mice treated with free DiD, DiD-PEG-Lipo, and DiD-ATF_24_-PEG-Lipo at predetermined time points after administration. (B) *Ex vivo* fluorescent images of the tumors and main organs excised from KU-19-19 tumor-bearing mice 60 h after treatment. (C) Quantified fluorescence intensity of free DiD, DiD-PEG-Lipo, and DiD-ATF_24_-PEG-Lipo in the spleen, liver and tumor. **P < 0.01; ***P < 0.001.

The KU-19-19 orthotopic bladder cancer model was successfully developed in nude mice. **Figure 4A** shows *in vivo* images of the KU-19-19 bladder cancer-bearing mice after an intravenous injection of free DiD or DiD-labeled liposomes. The free DiD group only showed fluorescence intensity in the liver and did not show accumulation in the tumor site, while the DiD-labeled liposomes showed significant fluorescence intensity in the liver and tumor site. The DiD signals of DiD-PEG-Lipo observed at 24, 48, and 60 h were weaker than those of DiD-ATF_24_-PEG-Lipo. In the DiD-ATF_24_-PEG-Lipo group, the fluorescence intensity at 48 h after injection was the strongest among the three time points. The *ex vivo* organs and tumors are shown in **Figure 4B and 4C**; free DiD, DiD-PEG-Lip, and DiD-ATF_24_-PEG-Lipo mainly accumulated in the liver and spleen, and the DiD fluorescence in the tumors in the DiD-ATF_24_-PEG-Lipo group was stronger than that in the DiD-PEG-Lipo group.

### *In vitro* cytotoxicity studies

**Figure 5:**
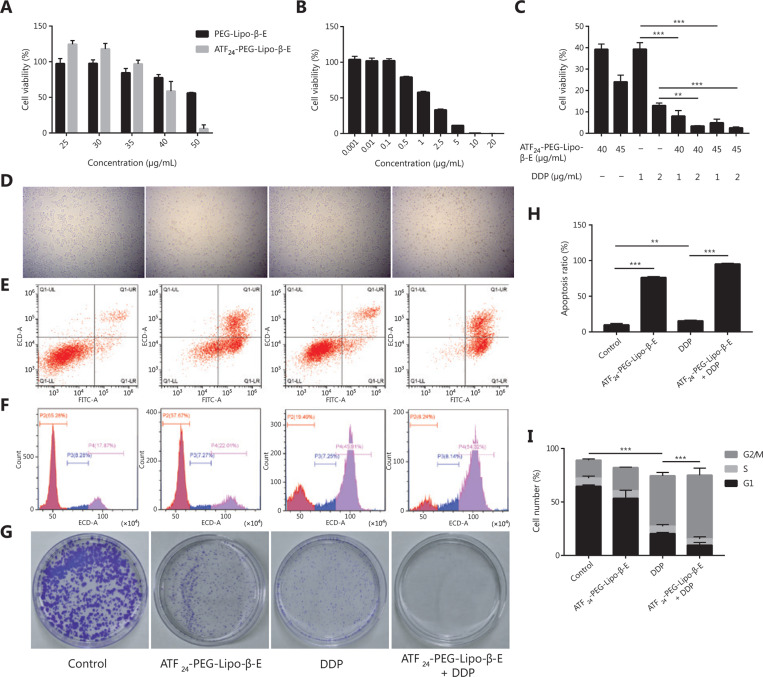
ATF_24_-PEG-Lipo-β-E and DDP synergistically inhibit the viability and proliferation of KU-19-19 cells. (A) The cells were treated with different concentrations of PEG-Lipo-β-E and ATF_24_-PEG-Lipo-β-E. (B) The cells were treated with different concentrations of DDP. (C) The cells were treated with ATF_24_-PEG-Lipo-β-E, DDP or their combination at the indicated concentration. (D) Images of KU-19-19 cells treated with ATF_24_-PEG-Lipo-β-E (45 μg/mL), DDP (1 μg/mL), or their combination for 48 h (20×). (E, H) KU-19-19 cells were treated with ATF_24_-PEG-Lipo-β-E (45 μg/mL), DDP (1 μg/mL), or their combination for 48 h. Cellular apoptosis was detected with Annexin V/PI double staining, followed by flow cytometry analysis. Representative images and quantification data are shown. (F, I) KU-19-19 cells were treated with ATF_24_-PEG-Lipo-β-E (45 μg/mL), DDP (1 μg/mL), or their combination for 48 h. Cell cycle analysis was performed following PI staining. Representative images and quantification data are shown. (G) KU-19-19 cells were incubated with ATF_24_-PEG-Lipo-β-E (8 μg/mL), DDP (0.2 μg/mL) or their combination for 12 days. Cellular colonigenic ability was examined by a colony-forming assay. **P < 0.01; ***P < 0.001.

As shown in **Figure 5A and 5B**, when the KU-19-19 cells were incubated with PEG-Lipo-β-E, ATF_24_-PEG-Lipo-β-E, or DDP for 48 h, the IC_50_ values were 50.49, 40.52, or 1.26 μg/mL, respectively. In the same concentration range, KU-19-19 cells treated with ATF_24_-PEG-Lipo-β-E exhibited higher cytotoxicity than those treated with PEG-Lipo-β-E. The enhanced efficiency of the ATF_24_-modified liposomes indicated that targeting of the nanoparticles into KU-19-19 cells through uPAR endocytosis increased the internalization of nanoparticles and, thus, led to a high percentage of β-E release. However, the enhanced killing effect was not obvious when compared with that of PEG-Lipo-β-E, which might be due to the small number of cells.

### ATF_24_-PEG-Lipo-β-E and DDP synergistically inhibit the viability and proliferation of bladder cancer cells

β-E has been shown to enhance DDP cytotoxicity in carcinomas of the bladder^43^. In this study, we investigated whether the combination of ATF_24_-PEG-Lipo-β-E and DDP had a synergistic effect. The viability percentages of the combination groups were significantly lower than that in each single drug group, indicating the greater toxicity efficacy of the combination therapy (**Figure 5C**). To further determine whether the improved cytotoxicity was the result of synergism or simply an additive effect by two drugs, the CI was calculated to quantitatively define synergism (CI < 0.90), an additive effect (CI = 0.90–1.10), or antagonism (CI > 1.10) in drug combinations^44,45^. The CI values of the three combination groups were below 0.90, indicating a synergistic effect of ATF_24_-PEG-Lipo-β-E and DDP (**Table 1**). The flow cytometry analysis indicated that DDP, in combination with ATF_24_-PEG-Lipo-β-E, caused an elevation in cellular apoptosis (**Figure 5D, 5E, and 5H**). We then assessed the effect of the combinatory treatment on cellular proliferation, so the cell cycles of cancer cells were first analyzed. The results showed that, compared with either single agent, the combination of ATF_24_-PEG-Lipo-β-E and DDP caused significant cell cycle arrest at the G2/M phase (**Figure 5F and 5I**). Consistent with this finding, the colony-forming ability of the KU-19-19 cells was significantly reduced by co-treatment of ATF_24_-PEG-Lipo-β-E and DDP, compared with those observed following treatment with either single agent treatment (**Figure 5G**)^46^. Together, the results indicated that the co-treatment of ATF_24_-PEG-Lipo-β-E and DDP had a synergistic effect in reducing cellular viability and proliferation in bladder cancer.

### ATF_24_-PEG-Lipo-β-E increased DDP cell apoptosis and cell cycle arrest by the caspase-dependent pathway and the Cdc25C/Cdc2/cyclin B1 signaling pathway

**Figure 6:**
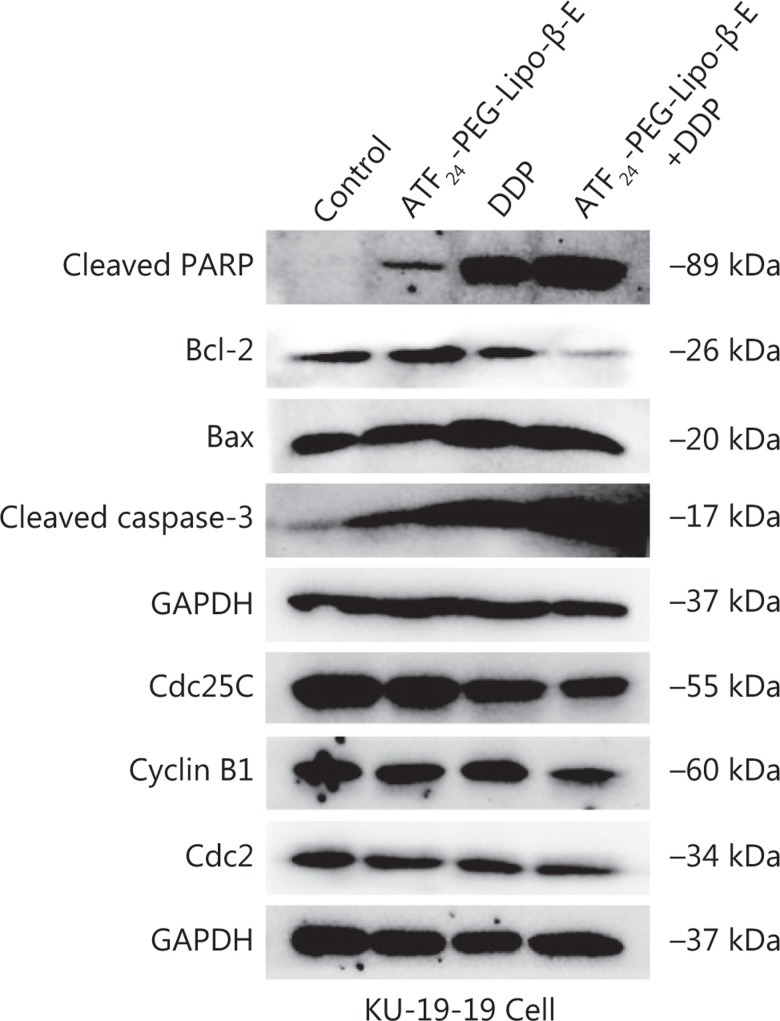
The expression of cell apoptosis- and cycle-related proteins following treatment with ATF_24_-PEG-Lipo-β-E (45 μg/mL), DDP (1 μg/mL), or their combination for 48 h.

Western blot was used to detect the levels of key proteins in the caspase-dependent pathway, and key proteins at the G2/M phase, in KU-19-19 cells treated with DDP combined with ATF_24_-PEG-Lipo-β-E for 48 h. The results showed that compared to the DDP treatment group, DDP combined with ATF_24_-PEG-Lipo-β-E decreased the expressions of Bcl-2, Cdc25C, Cdc2, and cyclin B1, but significantly increased the expressions of cleaved PARP, cleaved caspase-3, and Bax, when compared to the DDP treatment group (**Figure 6**)^47,48^.

### Combined treatment with ATF_24_-PEG-Lipo-β-E and DDP injection enhanced the antitumor effects in a bladder cancer xenograft model

**Figure 7:**
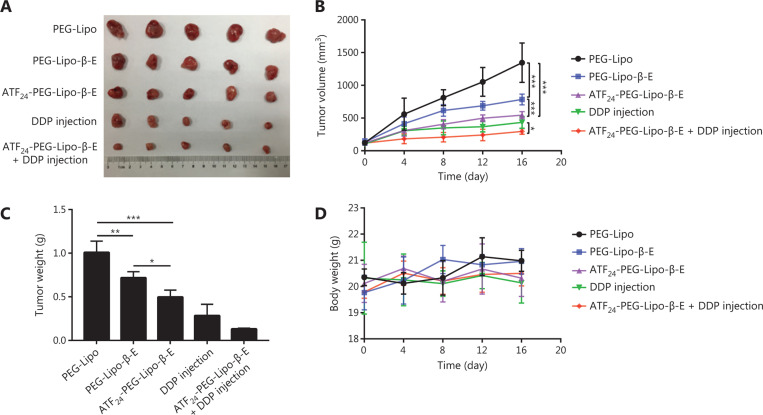
*In vivo* antitumor studies of different treatments alone or in combination using KU-19-19 bladder cancer tumor-bearing mice. (A) Representative images of dissected tumors treated with: PEG-Lipo-β-E, ATF_24_-PEG-Lipo-β-E, DDP injection, or ATF_24_-PEG-Lipo-β-E plus cisplatin (DDP) injection at a dose of 25 mg/kg for β-E and 5 mg/kg for DDP. (B) Tumor volume changes with different treatments alone or in combination. (C) Tumor weights at the end of the test. (D) Body weight change curves in different groups. *P < 0.05; **P < 0.01; ***P < 0.001.

As shown in **Figure 7A and 7B**, PEG-Lipo-β-E exhibited a high antitumor efficacy with an average TV of 784.77 ± 82.61 mm^3^. The antitumor efficacy of PEG-Lipo-β-E was augmented with an average TV of 544.95 ± 54.75 mm^3^ after conjugating the ATF_24_ peptide onto the liposomes, facilitated by its active targeting performance. Moreover, ATF_24_-PEG-Lipo-β-E further augmented the antitumor efficacy of the DDP injection through its sensitizing effect. The combination therapy resulted in profound tumor regression (74.18% decrease, as detected by TV), while the DDP injection treatment caused an approximate 58.75% decrease in the TV. Analyses of the tumor weights also confirmed that compared with the single agent treatments, the tumor weight was reduced by the drug combination (**Figure 7C**). As seen in **Figure 7D**, there was no significant decrease in body weight of mice during the treatment.

**Figure 8:**
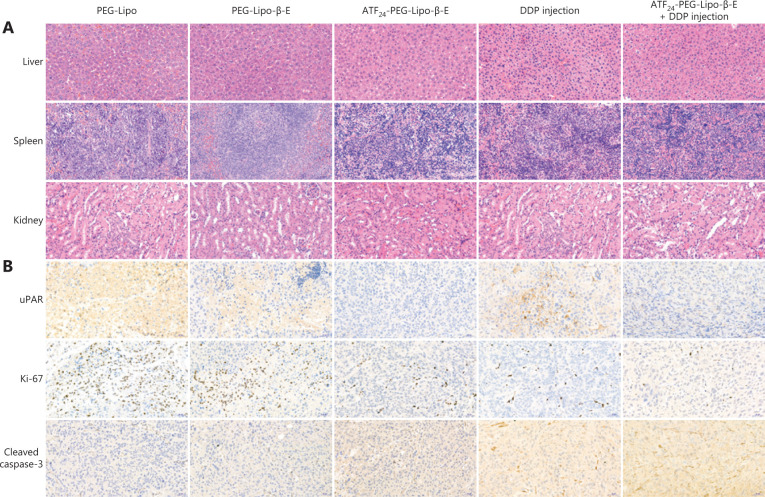
The safety of various formulations and the tumor progression level were evaluated by hematoxylin and eosin (H&E) staining and immunohistochemical staining. (A) H&E staining of the 3 organs including the liver, spleen, and kidneys (20×). (B) Tumor tissues were evaluated qualitatively to detect the urokinase-type plasminogen activator receptor, Ki-67, and cleaved caspase-3 expression using immunohistochemistry (20×).

Based on the H&E staining results (**Figure 8A**), we found that the hepatocytes and splenocytes were normal after drug treatment, and that the kidney exhibited the normal architecture of glomeruli and renal tubules without vascular expansion or congestion. No hepatorenal toxicity was observed by H&E staining in any group. Based on the immunochemical staining shown in **Figure 8B**, it was concluded that the ATF_24_-PEG-Lipo-β-E group did not show positive expression of uPAR, while the control group and PEG-Lipo-β-E group showed positive areas of uPAR. We also observed that the expression of uPAR in the combined group was lower than that in the DDP group. Proliferation is a key feature of the progression of tumors and is currently widely estimated to occur because of the Ki-67 nuclear antigen^49^. The expression of Ki-67 in the combined group was lower than that in the other three groups, and the control group had large positive areas of Ki-67. Caspase-3 is a type of protein that can be activated in apoptotic cells. We observed that the expression of cleaved caspase-3 in the combined group was higher than that in the other three groups. The control group had dense nuclei accumulation, but few cleaved caspase-3-positive spots. Overall, treatment with ATF_24_-PEG-Lipo-β-E and DDP injection prevented the KU-19-19 bladder cancer cells from further proliferation, and simultaneously induced apoptosis in cancer cells.

## Discussion

Over the past 30 years, drug delivery systems using liposomes have attracted attention due to their potential to improve the pharmacokinetics and biodistribution of free drugs and function as drug reservoirs. With small particle sizes, liposomes can passively target tumor tissues *via* the EPR effect. Liposomes can also be used to actively target tumors using ligands modified on liposomal surfaces^50–52^. However, the low delivery efficiency and limited tumor penetration of nanoparticle-based drug delivery systems are still considered an “Achilles heel” in tumor treatment^53^. Therefore, it is likely that tumor-targeting strategies enabling the nanoparticle carriers to simultaneously target the tumor microenvironment and tumor cells offer promising targeted drug delivery approaches.

Highly invasive bladder cancer does not have effective anticancer targets for clinical treatments. However, the overexpression of uPAR proteins has been observed in MIBC and several stromal cell types in the tumor microenvironment, providing a potential strategy for targeting MIBC^54^. Studies have indicated that the co-delivery of chemotherapeutic drugs using uPAR-targeted nanoparticles enhanced the therapeutic efficacy in triple negative breast cancer and pancreatic cancer animal tumor models. Currently, in clinical studies, β-E has been shown to be a promising adjunctive treatment, exerting a synergistic effect and improving patient outcomes in the treatment of cancer^55^. In this study, we developed uPAR-targeted liposomes carrying β-E to overcome the physical barrier of the stroma for the effective treatment of MIBC.

The uPAR-targeted liposome ingredient, DSPE-PEG_2000_-ATF_24_, was successfully synthesized by conjugating ATF_24_, the ATF peptide of the receptor-binding domain of uPA, a natural ligand of uPAR, with DSPE-PEG_2000_-COOH. ATF_24_-modified liposomes were successfully prepared with a high EE and a uniform size. The content of β-E in the ATF_24_ modified liposomes was equal to that of the commercial elemene injection. The FTIR and DSC analyses also demonstrated the formation of liposomes. Notably, the liposomes prepared in this study were characterized by TEM, which could not reflect the true appearance of the sample, and which is a shortcoming of this study, so the ATF_24_-PEG-Lipo-β-E needs to be characterized by cryo-TEM in subsequent studies.

The cellular uptake efficacy of liposomes was studied in the KU-19-19 cell line by confocal microscopy and UFLC. The fluorescence intensity after incubation with DiD-ATF_24_-PEG-Lipo was greater than that after incubation with DiD-PEG-Lipo. The cellular uptake as determined by UFLC was consistent with that in the fluorescence intensity analysis. Given the important role of uPAR in regulating matrix degradation, metastasis, and angiogenesis, we also compared the ability of liposomes to inhibit cell migration, by using wound healing and Transwell migration assays. KU-19-19 cells incubated with ATF_24_-PEG-Lipo-β-E produced the most effective results. Preincubation with excess free ATF_24_ peptide lowered the inhibitory effect of ATF_24_-PEG-Lipo-β-E. Notably, although the above results initially indicated that ATF_24_-PEG-Lipo-β-E had good targeting, the experimental design was not rigorous, and we did not use a scramble peptide sequence as control materials, which is a shortcoming in this study that needs to be addressed in subsequent research.

The biodistribution of ATF_24_-PEG-Lipo was also investigated in the KU-19-19 orthotopic bladder cancer model. Notably, fluorescence in the tumor tissues in the DiD-PEG-Lipo group gradually reduced from 24 to 60 h, while the fluorescence in the DiD-ATF_24_-PEG-Lipo group gradually increased up to 48 h, and then decreased until the end of the experiment. Additionally, the DiD signals in the liver and spleen were greater than those in the other organs, which might be due to capture by the reticuloendothelial system. The low accumulation of both liposomes in the heart also indicated no obvious heart toxicity using these liposomal delivery systems. Taken together, these results showed that ATF_24_ modification increased the tumor-specific delivery of ATF_24_-PEG-Lipo *in vivo*, likely resulting from the reduced drug flow back to circulation *via* an interaction between ATF_24_ in the liposomes and the uPAR expressed on the cell surface^56^.

DDP, which is among the most potent chemotherapeutic drugs, has activity against a variety of solid tumors^55^. Gemcitabine plus DDP is the neoadjuvant regimen of choice in many institutions treating patients with MIBC, but can be limited by severe side effects and drug resistance to the chemotherapeutic agents^57^. β-E enhanced the efficacy and reduced the toxicity of chemoradiotherapy and reversed the drug resistance. In the present study, our results showed that the combination of ATF_24_-PEG-Lipo-β-E and DDP significantly increased cell apoptosis, and arrested the cell cycle at the G2/M phase by the caspase-dependent pathway and the Cdc25C/Cdc2/cyclin B1 signaling pathway. Notably, the ATF_24_-PEG-Lipo-β-E showed a much higher level of apoptosis than DDP; however, the changes in the apoptosis-related proteins treated with ATF_24_-PEG-Lipo-β-E alone were not more obvious than those in the DDP treatment group. Apoptosis-related proteins (cleaved PARP, cleaved caspase-3, Bax, and Bcl-2) treated with ATF_24_-PEG-Lipo-β-E + DDP had a significant change compared with the DDP alone group. It might be possible that ATF_24_-PEG-Lipo-β-E did not primarily induce cell apoptosis *via* the mitochondrial pathway, but it could increase the sensitivity of tumor cells to DDP. The antitumor mechanism of ATF_24_-PEG-Lipo-β-E and the synergistic anti-tumor mechanism of the two drugs need to be further studied in the future.

The hepatorenal toxicity of DDP and the β-E liposomal formulations were important concerns in this study, because DDP and β-E are mainly metabolized by the liver and excreted by the kidneys, and the liposomes were found to be highly accumulated in the liver and spleen, as revealed by an *in vivo* imaging study. Therefore, the toxicity of the various formulations was assessed by histological tissue imaging of three organs (the liver, spleen, and kidneys) using H&E staining. There were no evident pathological abnormality in the three organs after the treatment with ATF_24_-PEG-Lipo-β-E and DDP injection. This finding confirmed the lack of toxicity to the major organs after combined treatment.

In the pathological analysis of tumor tissues, we detected three types of related cytokines, i.e., uPAR, Ki-67, and cleaved caspase-3. The low expression of Ki-67 indicated reduced growth of tumor cells. In addition, the high expression of cleaved caspase-3 in tumor sites indicated the occurrence of tumor cell apoptosis. Based on pathological analysis, we concluded that ATF_24_-PEG-Lipo-β-E and DDP injection effectively inhibited the proliferation of cancer cells, and induced cancer cells to undergo apoptosis. These results may be related to the decreased expression of uPAR.

## Figures and Tables

**Table 1:** The combination index values of ATF_24_-PEG-Lipo-β-E in combination with DDP

Dose (μg/mL)		CI
ATF_24_-PEG-Lipo-β-E	DDP	
40	1	0.94 ± 0.01
40	2	0.86 ± 0.06
45	1	0.88 ± 0.01
45	2	0.85 ± 0.04

The data are represented as the mean ± SD (*n* = 3). CI, combination index; ATF_24_-PEG-Lipo-β-E, ATF_24_ peptide-targeted liposome carrying β-elemene; DDP, cisplatin.

## References

[1] Bray F, Ferlay J, Soerjomataram I, Siegel RL, Torre LA, Jemal A (2018). Global cancer statistics 2018: globocan estimates of incidence and mortality worldwide for 36 cancers in 185 countries. CA Cancer J Clin..

[2] Yan H, Ren S, Lin Q, Yu Y, Chen C, Hua X (2019). Inhibition of UBE2N-dependent CDK6 protein degradation by miR-934 promotes human bladder cancer cell growth. FASEB J..

[3] Chen W, Sun K, Zheng R, Zeng H, Zhang S, Xia C (2018). Cancer incidence and mortality in China, 2014. Chin J Cancer Res..

[4] Dohn LH, Illemann M, Hoyer-Hansen G, Christensen IJ, Hostmark J, Litlekalsoy J (2015). Urokinase-type plasminogen activator receptor (upar) expression is associated with t-stage and survival in urothelial carcinoma of the bladder. Urol Oncol..

[5] Xia Y, Yuan M, Li S, Thuan UT, Nguyen TT, Kang TW (2018). Apigenin suppresses the IL-1beta-induced expression of the urokinase-type plasminogen activator receptor by inhibiting MAPK-mediated AP-1 and NF-kappab signaling in human bladder cancer T24 cells. J Agric Food Chem..

[6] Li Y, Sun B, Zhao X, Zhang D, Wang X, Zhu D (2015). Subpopulations of uPAR+ contribute to vasculogenic mimicry and metastasis in large cell lung cancer. Exp Mol Pathol..

[7] Seddighzadeh M, Steineck G, Larsson P, Wijkstrom H, Norming U, Onelov E (2002). Expression of UPA and UPAR is associated with the clinical course of urinary bladder neoplasms. Int J Cancer..

[8] Sidaway P (2015). Bladder cancer: uPAR expression indicates worse prognosis of urothelial carcinoma. Nat Rev Urol..

[9] Rao Malla R, Gopinath S, Alapati K, Gorantla B, Gondi CS, Rao JS (2013). Knockdown of cathepsin B and uPAR inhibits CD151 and alpha3beta1 integrin-mediated cell adhesion and invasion in glioma. Mol Carcinog..

[10] Hau AM, Leivo MZ, Gilder AS, Hu JJ, Gonias SL, Hansel DE (2017). mTORC2 activation is regulated by the urokinase receptor (uPAR) in bladder cancer. Cell Signal..

[11] Nguyen VD, Min HK, Kim CS, Han J, Park JO, Choi E (2019). Folate receptor-targeted liposomal nanocomplex for effective synergistic photothermal-chemotherapy of breast cancer in vivo. Colloids Surf B Biointerfaces..

[12] Jain RK, Stylianopoulos T (2010). Delivering nanomedicine to solid tumors. Nat Rev Clin Oncol..

[13] Sykes EA, Chen J, Zheng G, Chan WC (2014). Investigating the impact of nanoparticle size on active and passive tumor targeting efficiency. ACS Nano..

[14] Miller-Kleinhenz JM, Bozeman EN, Yang L (2015). Targeted nanoparticles for image-guided treatment of triple-negative breast cancer: clinical significance and technological advances. Wiley Interdiscip Rev Nanomed Nanobiotechnol..

[15] Nielsen BS, Rank F, Illemann M, Lund LR, Dano K (2007). Stromal cells associated with early invasive foci in human mammary ductal carcinoma in situ coexpress urokinase and urokinase receptor. Int J Cancer..

[16] Zhu L, Staley C, Kooby D, El-Rays B, Mao H, Yang L (2017). Current status of biomarker and targeted nanoparticle development: the precision oncology approach for pancreatic cancer therapy. Cancer Lett..

[17] Lee GY, Qian WP, Wang L, Wang YA, Staley CA, Satpathy M (2013). Theranostic nanoparticles with controlled release of gemcitabine for targeted therapy and MRI of pancreatic cancer. ACS Nano..

[18] Gao N, Bozeman EN, Qian W, Wang L, Chen H, Lipowska M (2017). Tumor penetrating theranostic nanoparticles for enhancement of targeted and image-guided drug delivery into peritoneal tumors following intraperitoneal delivery. Theranostics..

[19] Miller-Kleinhenz J, Guo X, Qian W, Zhou H, Bozeman EN, Zhu L (2018). Dual-targeting wnt and upa receptors using peptide conjugated ultra-small nanoparticle drug carriers inhibited cancer stem-cell phenotype in chemo-resistant breast cancer. Biomaterials..

[20] Zhai B, Zeng Y, Zeng Z, Zhang N, Li C, Zeng Y (2018). Drug delivery systems for elemene, its main active ingredient beta-elemene, and its derivatives in cancer therapy. Int J Nanomedicine..

[21] Zheng C, Cai X, Wu S, Liu Z, Shi Y, Zhou W (2014). Enhancing effect of beta-elemene emulsion on chemotherapy with harringtonine, aclacinomycin, and Ara-c in treatment of refractory/relapsed acute myeloid leukemia. Pak J Med Sci..

[22] Ma C, Zhou W, Yan Z, Qu M, Bu X (2016). Beta-elemene treatment of glioblastoma: a single-center retrospective study. OncoTargets Ther..

[23] Chang Z, Gao M, Zhang W, Song L, Jia Y, Qin Y (2017). Beta-elemene treatment is associated with improved outcomes of patients with esophageal squamous cell carcinoma. Surg Oncol..

[24] Li Z, Xie J, Peng S, Liu S, Wang Y, Lu W (2017). Novel strategy utilizing extracellular cysteine-rich domain of membrane receptor for constructing d-peptide mediated targeted drug delivery systems: a case study on Fn14. Bioconjug Chem..

[25] Zeng YY, Zeng YJ, Zhang NN, Li CX, Xie T, Zeng ZW (2019). The preparation, determination of a flexible complex liposome co-loaded with cabazitaxel and beta-elemene, and animal pharmacodynamics on paclitaxel-resistant lung adenocarcinoma. Molecules..

[26] Tefas LR, Sylvester B, Tomuta I, Sesarman A, Licarete E, Banciu M (2017). Development of antiproliferative long-circulating liposomes co-encapsulating doxorubicin and curcumin, through the use of a quality-by-design approach. Drug Des Devel Ther..

[27] Zeng Z, Zhou G, Wang X, Huang E, Zhan X, Liu J (2010). Preparation, characterization and relative bioavailability of oral elemene o/w microemulsion. Int J Nanomedicine..

[28] Chaves MA, Pinho SC (2019). Curcumin-loaded proliposomes produced by the coating of micronized sucrose: influence of the type of phospholipid on the physicochemical characteristics of powders and on the liposomes obtained by hydration. Food Chem..

[29] Dhoble S, Patravale V (2019). Development of anti-angiogenic erlotinib liposomal formulation for pulmonary hypertension: a QbD approach. Drug Deliv Transl Res..

[30] Zhuang CY, Li N, Wang M, Zhang XN, Pan WS, Peng JJ (2010). Preparation and characterization of vinpocetine loaded nanostructured lipid carriers (NLC) for improved oral bioavailability. Int J Pharm..

[31] Qiu Y, Yu Q, Liu Y, Tang J, Wang X, Lu Z (2018). Dual receptor targeting cell penetrating peptide modified liposome for glioma and breast cancer postoperative recurrence therapy. Pharm Res..

[32] Jiang K, Song X, Yang L, Li L, Wan Z, Sun X (2018). Enhanced antitumor and anti-metastasis efficacy against aggressive breast cancer with a fibronectin-targeting liposomal doxorubicin. J Control Release..

[33] Zhang Z, Cao H, Jiang S, Liu Z, He X, Yu H (2014). Nanoassembly of probucol enables novel therapeutic efficacy in the suppression of lung metastasis of breast cancer. Small..

[34] Ye LY, Hu S, Xu HE, Xu RR, Kong H, Zeng XN (2017). The effect of tetrandrine combined with cisplatin on proliferation and apoptosis of A549/DDP cells and A549 cells. Cancer Cell Int..

[35] Huang C, Yu Y (2017). Synergistic cytotoxicity of β-elemene and cisplatin in gingival squamous cell carcinoma by inhibition of STAT3 signaling pathway. Med Sci Monit..

[36] Grahovac J, Srdić-Rajić T, Francisco Santibañez J, Pavlović M, Čavić M, Radulović S (2019). Telmisartan induces melanoma cell apoptosis and synergizes with vemurafenib in vitro by altering cell bioenergetics. Cancer Biol Med.

[37] Li X, Wang G, Zhao J, Ding H, Cunningham C, Chen F (2005). Antiproliferative effect of beta-elemene in chemoresistant ovarian carcinoma cells is mediated through arrest of the cell cycle at the G2-M phase. Cell Mol Life Sci..

[38] Naletova I, Cucci LM, D’Angeli F, Anfuso CD, Magri A, La Mendola D (2019). A tunable nanoplatform of nanogold functionalised with angiogenin peptides for anti-angiogenic therapy of brain tumours. Cancers..

[39] Cao M, Long M, Chen Q, Lu Y, Luo Q, Zhao Y (2019). Development of beta-elemene and cisplatin co-loaded liposomes for effective lung cancer therapy and evaluation in patient-derived tumor xenografts. Pharm Res..

[40] Jin F, Wu Z, Hu X, Zhang J, Gao Z, Han X (2019). The PI3K/Akt/GSK-3β/ROS/eIF2B pathway promotes breast cancer growth and metastasis via suppression of NK cell cytotoxicity and tumor cell susceptibility. Cancer Biol Med.

[41] Meng L, Chu X, Xing H, Liu X, Xin X, Chen L (2019). Improving glioblastoma therapeutic outcomes via doxorubicin-loaded nanomicelles modified with borneol. Int J Pharm..

[42] Zhai B, Wu Q, Wang W, Zhang M, Han H, Li Q (2020). Preparation, characterization, pharmacokinetics and anticancer effects of pegylated β-elemene liposomes. Cancer Biol Med.

[43] Li QQ, Wang G, Liang H, Li JM, Huang F, Agarwal PK (2013). β-elemene promotes cisplatin-induced cell death in human bladder cancer and other carcinomas. Anticancer Res..

[44] Chou TC (2006). Theoretical basis, experimental design, and computerized simulation of synergism and antagonism in drug combination studies. Pharmacol Rev..

[45] Chou TC (2010). Drug combination studies and their synergy quantification using the Chou-Talalay method. Cancer Res..

[46] Cheng H, Ge X, Zhuo S, Gao Y, Zhu B, Zhang J (2018). Beta-elemene synergizes with gefitinib to inhibit stem-like phenotypes and progression of lung cancer via down-regulating EZH2. Front Pharmacol..

[47] Li QQ, Lee RX, Liang H, Zhong Y, Reed E (2013). Enhancement of cisplatin-induced apoptosis by beta-elemene in resistant human ovarian cancer cells. Med Oncol..

[48] Li QQ, Wang G, Huang F, Li JM, Cuff CF, Reed E (2013). Sensitization of lung cancer cells to cisplatin by beta-elemene is mediated through blockade of cell cycle progression: antitumor efficacies of beta-elemene and its synthetic analogs. Med Oncol..

[49] Chen C, Zhang Y, Huang Z, Wu J, Huang W, Zhang G (2019). Decrease in the Ki67 index during neoadjuvant chemotherapy predicts favorable relapse-free survival in patients with locally advanced breast cancer. Cancer Biol Med..

[50] Allen TM, Cullis PR (2004). Drug delivery systems: entering the mainstream. Science..

[51] Wilhelm S, Tavares AJ, Dai Q, Ohta S, Audet J, Dvorak HF (2016). Analysis of nanoparticle delivery to tumours. Nat Rev Mater..

[52] Wicki A, Witzigmann D, Balasubramanian V, Huwyler J (2015). Nanomedicine in cancer therapy: challenges, opportunities, and clinical applications. J Control Release..

[53] Bai S, Ma X, Shi X, Shao J, Zhang T, Wang Y (2019). Smart unimolecular micelles-based polyprodrug with dual redox stimuli-response for tumor microenvironment: enhanced in vivo delivery efficiency and tumor penetration. ACS Appl Mater Interfaces..

[54] Buchler P, Reber HA, Tomlinson JS, Hankinson O, Kallifatidis G, Friess H (2009). Transcriptional regulation of urokinase-type plasminogen activator receptor by hypoxia-inducible factor 1 is crucial for invasion of pancreatic and liver cancer. Neoplasia..

[55] Zhai B, Zhang N, Han X, Li Q, Zhang M, Chen X (2019). Molecular targets of beta-elemene, a herbal extract used in traditional chinese medicine, and its potential role in cancer therapy: a review. Biomed Pharmacother..

[56] Dai W, Yang F, Ma L, Fan Y, He B, He Q (2014). Combined mTOR inhibitor rapamycin and doxorubicin-loaded cyclic octapeptide modified liposomes for targeting integrin alpha3 in triple-negative breast cancer. Biomaterials..

[57] Meleis L, Moore R, Inman BA, Harrison MR (2020). Retrospective analysis of the efficacy and safety of neoadjuvant gemcitabine and cisplatin in muscle-invasive bladder cancer. J Oncol Pharm Pract..

